# The role of science in a crisis: Talks by political leaders during the COVID-19 pandemic

**DOI:** 10.1371/journal.pone.0282529

**Published:** 2023-03-24

**Authors:** Enzo Loner, Eliana Fattorini, Massimiano Bucchi

**Affiliations:** Department of Sociology and Social Research, University of Trento, Trento, Italy; Manaaki Whenua - Landcare Research, NEW ZEALAND

## Abstract

During the COVID-19 pandemic, science has been prominently featured in institutional communication and political agendas as never before. Governments substantially relied on scientific experts to analyze pandemic trends, develop anti-COVID-19 vaccines and adopt containment strategies. In this paper, we analyze speeches by three political leaders–Boris Johnson (Prime Minister, UK), Sergio Mattarella (President of the Republic, Italy), and Ursula von der Leyen (President, European Commission)–between February 20, 2020, and February 20, 2022, to identify how science was addressed and framed. The results of the quantitative and qualitative exploration of the speeches highlight three main ways in which political leaders view science: *a national pride* narrative–i.e., science as an instrument and indicator of national pride and international standing of the country; *an ethical* narrative–i.e., science as an agent of social growth; an *integration* narrative–i.e., science as a driving force of both European integration and stronger collaboration between knowledge production and industry. The predominant narrative varies in relation to the political leaders’ different institutional contexts and roles.

## Introduction

From a Science and Technology Studies point of view, the COVID-19 pandemic has represented a global science communication challenge and an unprecedented opportunity to deepen the relationship(s) between science and society [1]. Indeed, during this health crisis, science has been at the core of citizens’ daily life, political agendas, and institutional and media communication as never before. Furthermore, given that the SARS-COV-2 virus was new and thus unknown to the scientific community, it has represented an occasion "to see the scientific enterprise as it proceeds in real time" [2].

The first cases of the novel coronavirus–officially named COVID-19 on February 11, 2020, by the World Health Organization [3]–were registered in China at the end of 2019. Then, from January 2020, other countries started to report confirmed infection cases. On January 30, 2020, the World Health Organization declared first the virus a "Public Health Emergency of International Concern" [4] and then on March 11, 2020, given its global spread, it became a pandemic [5]. Political leaders have adopted different strategies to manage COVID-19-related issues and communicate country-specific priorities [6]. Besides other topics, during this pandemic period, political actors all around the globe addressed science-related issues in their speeches in different ways. As highlighted by [7], during periods of major threats–such as the COVID-19 pandemic–governments increasingly ask for scientific expertise to help them understand the crisis and design interventions, leading to a need to adopt evidence-based policymaking. However, due to the novelty of the virus, "in the early days of the pandemic, decisions about public-health measures were hampered by a lack of knowledge about how the virus is transmitted" [8]. A period in which science was particularly uncertain and in which the involvement of scientific experts as policy advisors has produced two concomitant results [7]: first, science and scientists have benefited from increased public confidence; second, science-related information has been increasingly politicized.

While the virus was declared a pandemic in March 2020, COVID-19-related topics were already present in political leaders’ speeches in early February. Accordingly, in our analysis, the speeches were collected starting from February 20, 2020. Our paper analyses both quantitatively and qualitatively the speeches of three leading political actors–i.e., Boris Johnson (Prime Minister of the United Kingdom), Sergio Mattarella (President of Italy), and Ursula von der Leyen (President of the European Commission)–held between February 20, 2020, and February 20, 2022. To study science-related narratives during the pandemic, we focused on these three political leaders because of the pandemic’s impact and the specific COVID-19 response implemented by the countries and supranational institutions considered. The British government was the first to approve and distribute anti-COVID-19 vaccines to its citizens.

Moreover, Boris Johnson–initially sceptical regarding implementing strict containment measures–changed his COVID-19 strategy by adopting a more scientific-oriented approach. Italy, instead, was the first Western country to implement restrictive measures to reduce the spread of COVID-19 –given that it was the first Western country to be hit by the virus. In this context, Sergio Mattarella has been a valued actor during the pandemic. Among the supranational institutions that intervened after the onset of the health emergency, the European Commission (EC)–chaired by Ursula von der Leyen–has been on the frontline communicating the views of the EC as EU’s. Besides measures introduced to support countries’ economies, von der Leyen has worked to mediate between the (heterogeneous) Member States’ approaches in dealing with emergencies.

The selected actors also play different political roles (and cultures): Johnson and Mattarella are country-level leaders, even with different roles. At the same time, von der Leyen leads an institution at the supranational level. These three actors play different roles in strictly political terms and significantly represent three different COVID-19 crisis management strategies in Europe and three different styles of communication in Western Europe. Specifically, two main aims guided our descriptive analysis of their speeches: first, to identify the main narratives about science that emerge from the selected political leaders’ speeches; second, to understand whether and how these narratives might differ. Awareness of science-related narratives in political discourses is fundamental both to identify how science is linked to key political narratives and to understand how science and scientific expertise have influenced–and arguably will influence–broader policy decisions.

### Science and politics during the COVID-19 pandemic

How political leaders have publicly addressed COVID-19-related topics can influence both levels of trust and the type of citizens’ response [7]. Indeed, as [9] highlighted, people’s attitudes and behaviours can be influenced by how the COVID-19 crisis has been framed in scientific discussions, media and social media communication, and political speeches. Also, trust in key actors is fundamental to enhancing citizens’ adherence to restrictive and protective measures embracement [10, 11] and increasing the willingness to be vaccinated [12, 13]. Especially in highly uncertain and complex contexts–like the COVID-19 crisis–attitudes and behaviours can be shaped by relying on institutions, policymakers, and scientific experts [14].

Starting in early 2020, the link between science and politics has strengthened and become more explicit. Indeed, during the pandemic, scientists have been asked for their specialist support to design, orient, and implement measures to manage the spread of the virus. Dedicated scientific advisory boards were created [15] to allow governments implementation of evidence-based strategies [16]. Besides politicians, science and scientists have thus played a key role. However, with the onset of the COVID-19 crisis, public-health policies were introduced without knowledge about the virus. This illustrates how scientific experts and political actors had to progressively re-calibrate measures introduced considering new knowledge advancements [7, 8]. In such an uncertain situation, "the ability of heads of governments and global health authority figures to communicate publicly on the impact of COVID-19 and the measures taken to mitigate risks are critical" [6]. Furthermore, politics and science had to deal with the widespread demand for open and transparent information [17, 18].

Especially in the first stages of the pandemic, scientists–not only from the health and life sciences fields but also experts from the social sciences [19]–had to provide to governments evidence in a strict time frame, working in an uncertain, fast-changing environment. Leading political leaders had to make decisions in a period in which there was high scientific uncertainty–e.g., regarding COVID-19 severity and transmissibility–and daily debates among experts on the best strategy to manage the contagion [20, 21]. In this complex scenario, political leaders appealed to their nation by emphasizing their support for science (and scientists) and the need to rely on it (them). Precisely, in Western countries [22], technocratic-oriented messages about the virus and "follow the science" claims characterized governmental and scientific communication during the first phase of the pandemic. As highlighted by [23], appealing to science–and relying on scientific experts–can reinforce policy strategies and increase citizens’ policies’ acceptance. As [24] pointed out, since the onset of the pandemic, citizens experienced a polarized political debate regarding the COVID-19 health issue, its gravity, and the best strategy to manage it. Furthermore [25], highlighted that leaders’ reputations–built before the outbreak of a crisis, such as the COVID-19 pandemic–can affect citizens’ trust and willingness to follow institutional dispositions. During an emergency, public confidence can also be reduced by communications or actions that emphasize a lack of coordination, agreement, and transparency among scientific experts and political leaders [26].

Many studies have analyzed the communication of political actors during the COVID-19 pandemic, but the representation of science in official political discourses was rarely explored. In their semiotic analysis of the speeches of Italian, French, and Spanish presidents during the pandemic [27], concluded that the political leaders discursively used the relationship between science and politics to strengthen or justify the strategies chosen. [22] analyzed the Dutch Prime Minister’s press conferences and the related Twitter responses in the first stage of the pandemic. Their research showed that in these official statements, scientific experts were presented as entirely reliable and competent to propose COVID-19-related measures, thus deserving to be blindly trusted. However, social media extensively criticized the government’s incapacity to acknowledge and communicating uncertainty among scientists. According to [28]–who analyzed 29 political leaders’ statements made during the early phase of the pandemic outbreak–despite the different communication strategies adopted by political actors in their speeches, most of them share a common characteristic, i.e., nationalism. Indeed, scientific experts are mainly presented in leaders’ speeches from a national point of view and are often invited to institutional press conferences. Given the rise of support for populist ideologies in Europe in the last decade [29, 30] and the increased politicization of science during the COVID-19 pandemic [31], other studies specifically analyzed the role played by science in populist narratives about the health crisis. In their research about governmental responses to the pandemic and their impact on citizens’ behaviors [32], highlighted that populist governments were more likely to implement popular short-term policies and share anti-scientific positions belittling the severity of the crisis–thus increasing citizens’ likelihood of non-compliance with preventive measures and of minor threat perceptions. These results are in line with previous literature on populists’ anti-scientific attitudes [33–35]–especially regarding criticism towards scientific institutions and scientific experts [36]–and their influence in decreasing citizens’ compliance towards public health measures [37].

Given the relevance of science and politics during the pandemic period and the limited research specifically oriented to analyzing how science is represented and communicated in political actors’ speeches, our paper aims to give a preliminary and descriptive contribution to this literature. Our analysis focuses on three cases: the United Kingdom, Italy, and the European Commission.

### The contexts

#### The United Kingdom: Boris Johnson

We selected the United Kingdom (UK) case for two main reasons. First, the UK was the first European country to approve the new anti-COVID-19 vaccines and distribute them to its citizens [38]. Second, Prime Minister Boris Johnson’s strategy to manage the pandemic changed from the initial "mitigation" approach to one more in line with the World Health Organization’s recommendations [8, 39].

In 2019, Conservative prime minister Boris Johnson was elected Theresa May’s successor. During her legislation, May failed to present a deal for exiting the European Union (also known as "Brexit")–and thus the results of the 2016 referendum–that could be approved by the parliament. Instead, since 2019 Johnson has focused his electoral campaign and mandate on the slogan "Get Brexit done" [8]. The official Brexit was achieved on January 31, 2020, and British citizens still perceive the topic as highly divisive. With the onset of the COVID-19 pandemic, the UK government decided to adopt a strategy that was not aligned with the World Health Organization recommendations. Indeed, at the early stages of the health crisis, Johnson introduced measures only to slow the increase in the COVID-19 infection rate to (potentially) reach herd immunity through natural infection. In this initial phase, during which no vaccines were available, Johnson evaluated the implementation of non-pharmaceutical interventions–such as quarantine and social distancing–mainly for older adults and vulnerable individuals [40]. On March 23, 2020, the UK government decided to change strategy and introduce lockdown throughout the nation: the forecast models showed a drastic increase in the mortality rate if the "mitigation" strategy were pursued [41]. Soon after the national lockdown, Johnson tested positive for COVID-19. In this context, the governmental communication appeared to be vague, confusing, and at times contradictory [42], which led to several U-turns that compromised its credibility [43].

The scientific community criticized the delayed implementation of restrictive measures to reduce the spread of the virus–e.g., national lockdown and border closure. As [8] highlighted, this constituted "the first of several tensions between what ’the science’ seemed to recommend and the apparent constraints of other policy considerations".

#### Italy: Sergio Mattarella

We included in our analysis of leaders’ speeches those of the president of the Italian Republic, Sergio Mattarella. Italy was the first Western country hit by COVID-19, and Mattarella has been an ever-present figure during the pandemic and the succession of governments.

Italy has registered a rapid and constant increase in infections and mortality rates since the end of February 2020, especially in the Northern regions [44]. The "Italian model" to fight the virus–the first package of measures adopted in Europe to manage the spread of the virus–provided a first reference model for other countries that faced the COVID-19 threat [7, 45].

Between 2020 and 2021, two succeeding Presidents of the Council were Giuseppe Conte and Mario Draghi. The Italian government, led by Conte, implemented the first national lockdown on March 9, 2020, and on April 26, 2020, extended the lockdown and mobility restrictions further. Health authorities and scientific experts mainly supported these decisions [46]. Furthermore, the decision-making process in the COVID-19 context has been supported by a dedicated task force–i.e., Technical-Scientific Committee (TSC). In January 2021, the president of the Council, Giuseppe Conte, and his government did not reach the absolute majority in the confidence vote. Thus, the president of the Republic, Sergio Mattarella, had to intervene to find a new political equilibrium. After consultation with the political parties involved, he officially gave Mario Draghi the mandate to form a new government. Unlike his predecessor, Draghi adopted a low-profile communication strategy, limiting the frequency of his public appearances [47].

Sergio Mattarella has been a fundamental actor since the onset of the COVID-19 crisis. Indeed, despite the end of his seven-year mandate on February 3, 2022, he was asked to remain in charge for a second seven-year mandate. This request was mainly due to the impossibility of finding a shared candidate as the new president of the Republic, considering his recognized ability to overcome the deep political divisions among parties. These led to Mattarella’s re-election by the Italian parliament on January 29, 2022. During the pandemic, Mattarella received renewed political confidence and increased trust from Italian citizens [48]. In the COVID-19 context, Mattarella––played a more "ceremonial role" [49]–given his institutional role in the parliamentary Republic–and his public appearances were aimed at providing institutional guidance and communicating a sense of unity for the country.

#### The European Commission: Ursula von der Leyen

In managing the COVID-19 pandemic, also international institutions played a key role. In the case of the European Union (EU), the European Commission (EC)–guided by Ursula von der Leyen–has actively supported the Member States in managing the pandemic.

In late February 2020, COVID spread in Italy and rapidly started its diffusion in other European countries. In March 2020, EC President von der Leyen established a "corona response team" [50].

As [51] highlighted, the COVID-19 crisis has clearly shown and stressed difficulties both at a country level–in terms of readiness and emergency response–and at a supranational level–in the capacity to coordinate and manage healthcare supplies and other public goods. However, the EU has specifically implemented several strategies to help EU countries face the virus and its consequences. Indeed, the EU invested in facilitating Member States’ purchasing of health-related products [52], provided financial aid to support workers and the recovery of the economy [44], and adopted the EU4Health program (2021–2027) to increase the EU crisis readiness. Notably, the EU adopted a centralized approach [53] to manage the purchase of anti-COVID-19 vaccine doses and promote their development in the union. In this context, the EC represented all Member States in negotiations over vaccine purchase deals with the pharmaceutical companies involved, securing lower prices and preventing more affluent Member States from getting all available vaccines in spite of financially weaker countries. Furthermore, in line with the request by the World Health Organization for a global response against COVID-19, the EC–in collaboration with the European External Action Service–produced daily reports on COVID-19-related data accessible to all Member States.

However, the EC’s efforts to manage and distribute anti-COVID-19 vaccines have not been free from criticism–especially from Germany. Indeed, there were several delays in the production and delivery of the agreed doses in the Member States. In February 2021, Ursula von der Leyen intervened, admitting that the EC underestimated the timing of vaccine production and distribution [53]. However, the early EU response to COVID-19 was mainly slowed and weakened by Member States’ nationalistic approaches to the crisis [54]–aimed at securing exiguous health-related resources for their citizens–and by legal issues regarding cross-national COVID-19 tracking [55]–a situation worsened by the exiting of the United Kingdom from the EU.

## Data and methods

In the present work, we analyze all the speeches by Ursula von der Leyen, Boris Johnson and Sergio Mattarella between February 20, 2020, and February 20, 2022. Data are available for download from the following websites: https://ec.europa.eu/commission/presscorner/detail/en/ (von der Layen), https://www.ukpol.co.uk (Johnson), and https://www.quirinale.it/ricerca/discorsi (Mattarella).

Data collection involved three steps: 1) Downloading the speeches: 115 for von der Leyen, 94 for Johnson, and 107 for Mattarella (Table 1). The total number of texts collected was 316. 2) Translating Mattarella’s discourses from Italian into English. 3) Select the fragments about science. The keywords used to extract the excerpts were: science/sciences, scientific and scientist/scientists. Each excerpt included the keyword and the five words preceding and following it. The fragments summed to 359: 102 for von der Leyen, 133 for Johnson and 124 for Mattarella.

**Table 1:** Speeches delivered by the three leaders and fragments regarding science (2020–22).

	Boris Johnson	Sergio Mattarella	Ursula von der Leyen
N. of speeches	94	107	115
Words by speech:			
• Average	932.3	1142.0	1193.5
• Min	52	123	164
• Max	5711	3613	3286
Frames about science (n)	133	124	102
*Ratio frames/speeches*	*1*.*41*	*1*.*16*	*0*.*89*
Frames science/s (%)	37.6	53.2	59.8
Frames scientist/s (%)	33.8	12,1	25.5
Frames scientific (%)	28.6	34.7	14.7
*Total (%) *	*100*	*100*	*100*

The preparation and cleaning of the excerpts followed the steps suggested by [56]: lowercasing and punctuation removal; removal of English stop words; stemming (to reduce all the words to their common root); and construction of the speech’s corpus, i.e., the collection of documents.

Since the corpus of documents included only official speeches, text cleaning was more straightforward than the cleaning usually necessary to prepare texts downloaded from social networks. For this purpose, we used the stem_snowball procedure of the corpus library [73] and the R language [67]. Stopwords were excluded from co-occurrence networks. In detail, we removed the stopwords included in the two sets stopwords-iso (1298 stopwords) and nltk (179 stopwords) of the R library stopwords [74]. In addition, a few words that often occur in courtesy phrases were removed: ladies, gentlemen, friends, address and fellow. Removing the stopwords allowed us to highlight the underlying language’s structure better. Consequently, text cleaning made it possible to focus better on the most critical aspects of the leaders’ speeches. The procedure’s correctness was also tested with a qualitative inspection of the texts.

The analysis focused on a mixed-method procedure that bridges qualitative and quantitative text analysis. The method adds a qualitative examination of the speeches to the quantitative analysis. The quantitative analysis increases efficiency by allowing time and cost savings compared to qualitative research [57]. Moreover, it will enable using more extensive data sets. Further, it contributes to increasing the representativeness of the study and reinforces the rigour of the results. Finally, qualitative assessment helps check the consistency of the results obtained through quantitative analysis.

To conduct a qualitative inspection of the fragments, we linked them back to the discourses from which they were extracted. In this way, it was possible 1) to confront the results of the quantitative analysis with the evidence of the speeches and 2) to extrapolate fragments to understand better and interpret the networks and clusters of words.

Ultimately, both methodologies contribute to a better understanding of the structure of the texts [58].

The fragments were tokenized—i.e., divided into words—before being analyzed. Subsequently, we identified bigrams. Bigrams are sequences of two consecutive terms. As bi-grams are two adjacent words, they represent, in practice, the same concept as co-occurrences. According to [59], we define co-occurrence as:

The adjacency of two word forms in sentence formation. For instance, in "John kicked the ball", there are three pairs of adjacent word forms, namely "John kicked", "kicked the", and "the ball". A word co-occurrence network thus can be represented by an undirected graph G = (V, E). V is the set of vertices representing all the different word forms in the language data. E, on the other hand, is the set of edges representing all different adjacency relations of the word forms in sentence formation. Therefore, two vertices u, v ∈ V are joined by an edge e ∈ E if the two corresponding word forms are adjacent within at least one sentence. According to this definition, we can extract all the different word-form bigrams in sentence formation from the authentic language data and convert this set of bigrams into the word co-occurrence network.

Co-occurrences (and bigrams) allow for a topological reconstruction of the characteristics of the speeches. The graphic representation of the networks built using the co-occurrences (between words) originates from social network analysis [60]. This method, also referred to as textual network analysis [58], allows for identifying the main themes covered in the speeches. The visual representation of the network helps study the structure of the discourses.

This method represents an attempt to link content analysis with network analysis. It focuses on the interactions between the nodes (the words) that make up the network [58]. In the network analysis language, words are the nodes (of the network). Moreover, relations, represented by the bigrams, i.e. how many times a word occurs before or after another, are the edges used to construct networks.

Furthermore, it helps extract the meanings from the speeches as clustering words highlighting the semantics of the narratives [61, 62].

The analysis of co-occurrence networks is appropriate for our work for at least three reasons. First, co-occurrences can be used to create a graphic representation of the concepts of science elaborated in the rhetoric of communication of each political leader. Second, the visual representation allows one to understand better the text’s structure and the main underlying ideas on which the narrative about science is based. Finally, it is possible to obtain quantitative measures from the networks. Not surprisingly, networks are used to study language from psycholinguistic research [63], but they have also recently been applied to other fields, such as the study of historical texts [64] or the communication of political leaders [65, 66].

All statistical analyses were performed with the R software [67].

## Results

Building upon our mixed methodology–which uses both quantitative and qualitative exploration of the speeches–we now turn to the results of our research (Table 1). This section presents the analysis of the discourses of Boris Johnson, Sergio Mattarella, and Ursula von der Leyen, delivered between February 20, 2020, and February 20, 2022.

### Boris Johnson: The representation of science as a national pride

**Fig 1:**
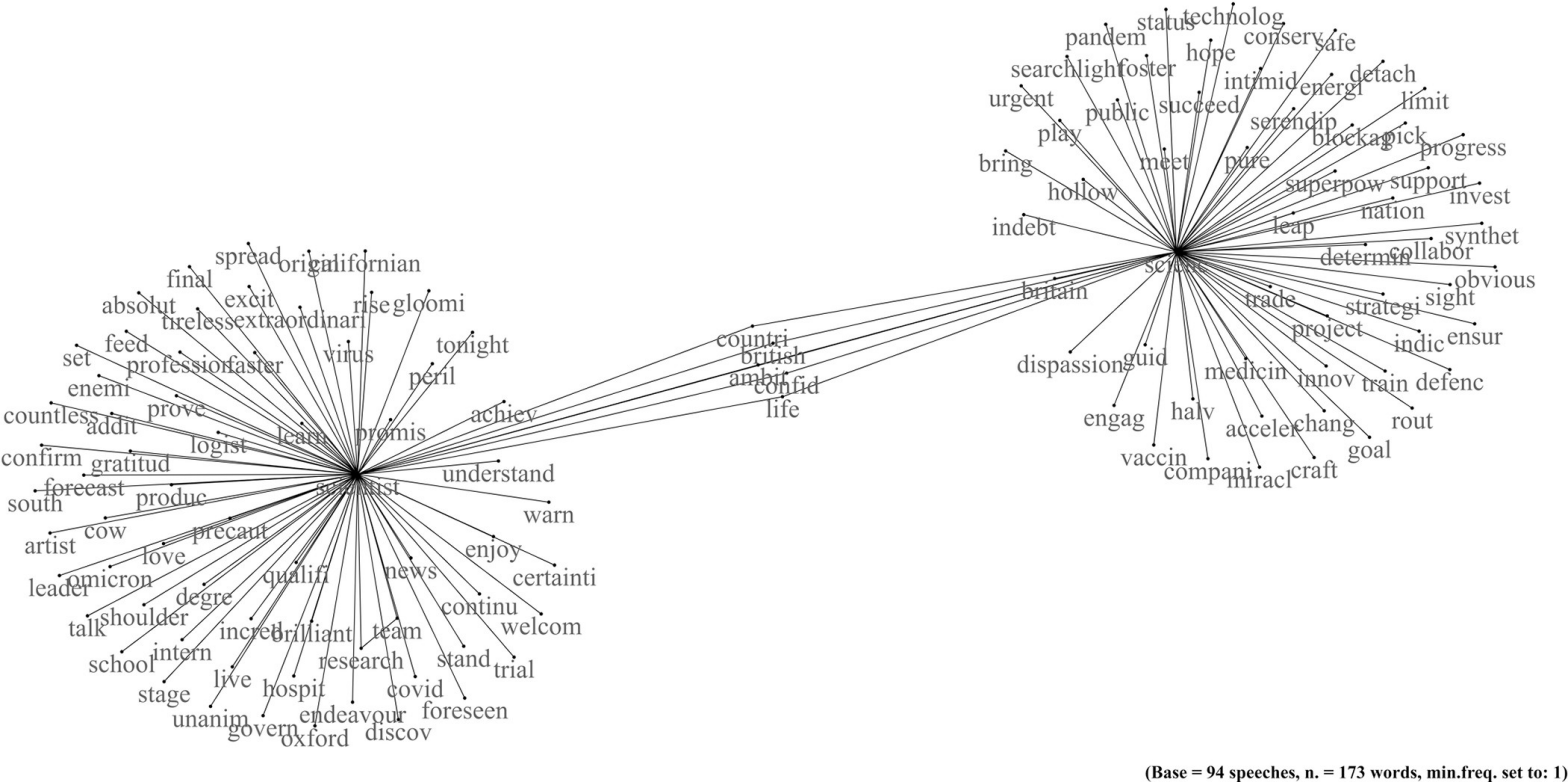
Johnson. Network of word co-occurrences about science.

Boris Johnson cited science 133 times in the 94 speeches delivered in the first two years of the pandemic (the ratio between the extracted fragments and the speeches delivered is 1.41). Therefore, science is a recurring topic in his communication. It is possible to obtain an overall picture of his view by observing the network of co-occurrences (Fig 1) and comparing it with some of the fragments extracted from the speeches (See Tables A1 and A2 in the S1 Appendix for some statistics about the texts and the network). The sub-network on the right contains the words that follow or precede "science". It, thus, includes the characteristics and aspects closest to science extracted from public communications. The sub-network on the left relates to scientists. Some terms, including "country" and "British", connect the two networks. This link demonstrates a close connection between science and scientists on one side and the country on the other within Johnson’s narrative.

The following fragment is an example of the first theme we have identified: the instrumental use of science to develop a national identity. In this excerpt, Johnson talks about the British scientists researching to fight the covid. The premier exalts the value of his country’s sciences. In doing so, he appeals to pride and, at the same time, to national identification:

We will build on the expertise and originality of our scientists, who have allowed Britain to contribute more to the global struggle against covid than any other comparable country, providing an object lesson in the value of British life sciences. (J, fragment n. 34).

Johnson skilfully links the image of the scientists to Great Britain. Therefore, the resulting idea is that the country contributed "more than any other" to fighting covid. Scientists are then evoked with a precise goal, as their success is evidence of the power of the United Kingdom. Hence, the citation of science and its actors is instrumental. The key (here and in other fragments) is using the words "our scientists", who appear—even if he does not say so directly—as the representatives "of our people" or "our heroes" and help heighten national pride.

The second theme is about the rhetoric of British power. In the following excerpt, the leader identifies a specific purpose: to have the UK regain its place as a scientific superpower.

"We want the UK to regain its status as a science superpower and, in so doing, to level up." (J, fragment n. 5).

The term "superpower" also emerges from the network of co-occurrences, as well as "nation" and "status". These words reinforce its use from an instrumental point of view to foster identification. Even financing science is associated with defence, which will serve "to engage with and help the rest of the world" (J, fragment n. 18). Therefore, the superpower narrative translates beyond the scientific sphere, including military power. In addition, this reference is also a way to propose a development model that will create "hundreds of high-skilled jobs across the country" (J, fragment n. 12). For Johnson, science will thus become the driving force of the country’s well-being. In the network of co-occurrences, this theme is represented, for instance, by the words "invest", "change", "progress", "ensure", and "train".

The third and final theme is the guide of science. In the network, it emerges from words such as "guide", "goal", and "strategic". During the pandemic, the premier stated that "we must, and we will be guided by the science" (J, fragment n. 47). However, elsewhere, he reiterates that politics and the government "have a role in making demands, explicitly framing the challenges we hope that science can meet" (J, fragment n. 17). Consequently, a representation of science as an instrument of politics emerges. Science is a means to solve problems, create jobs and contribute to the country’s green revolution, given its "Promethean faith in new green technology" (J, fragment n. 8).

In Johnson’s view, science is supported by both public and private resources, as evidenced by the following example:

"The whole experience of the Covid pandemic is that the way to fix the problem is through science and innovation, the breakthroughs and the investment made possible by capitalism and free markets (J, fragment n. 8)."

Science is, then, a key to the success of the country. At the same time, however, if it is made possible by capital and the free market, the latter and the government will set the objectives it has to achieve.

### Sergio Mattarella: The ethical representation of science

**Fig 2:**
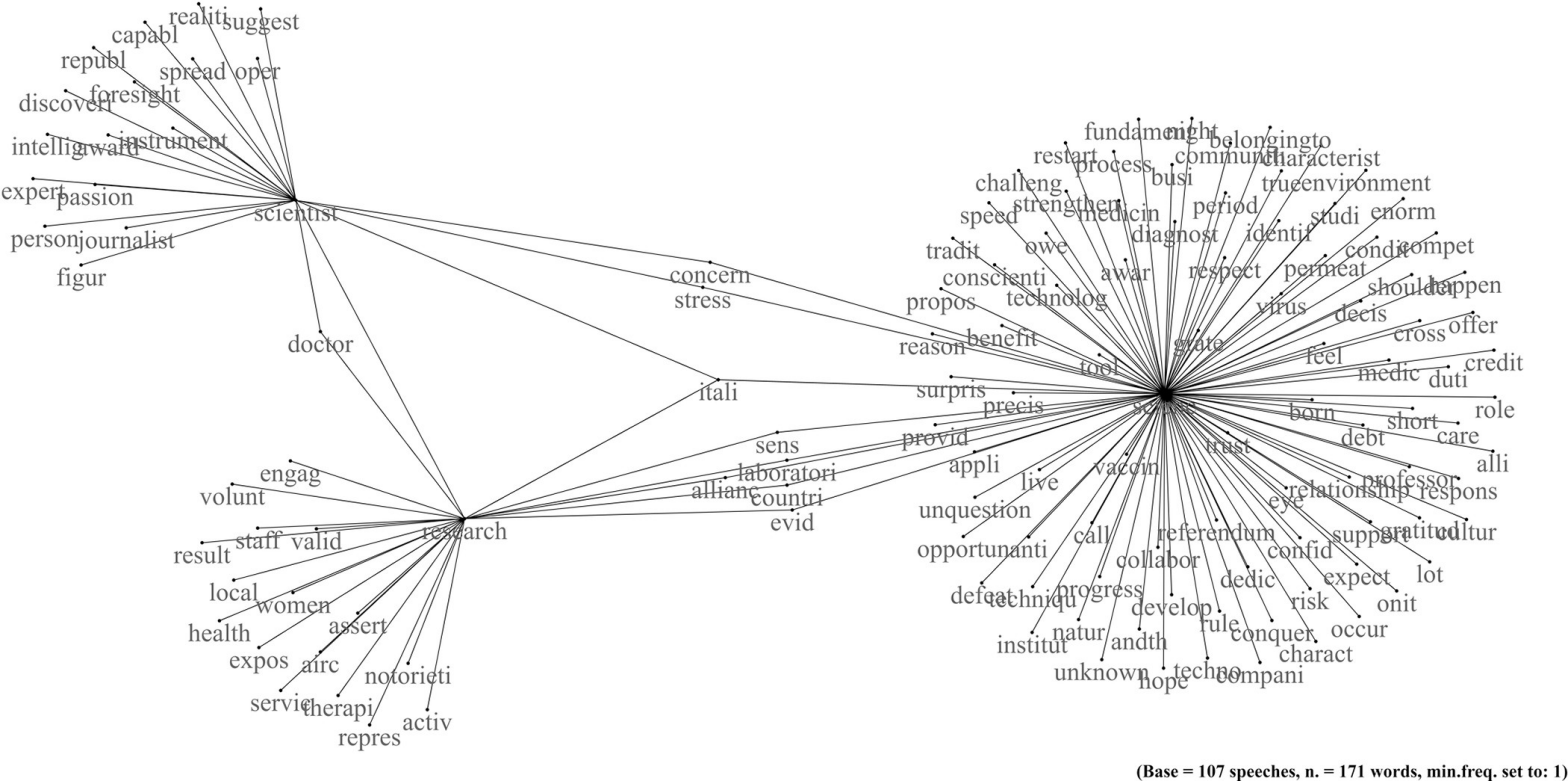
Mattarella. Network of word co-occurrences about science.

Mattarella cited science 124 times in his speeches delivered during the pandemic, with a fragment/speech ratio of 1.16 (Table 1). The network of co-occurrences includes three sub-networks (Fig 2). The first contains science-related words, while the other two concern research and scientists. What binds the three sub-networks together is the word "Italy". As for Johnson, there is a union around the country. However, other elements make the president of the Italian Republic’s discourses different from the British Prime Minister’s. For example, he mentions that Italian scientists get recognition for their discoveries; but he also notes that they operate in research institutes worldwide. In the network relating to scientists, the words: "passion", "discovery", "expert", and "award" express this condition (Fig 2). The following fragment gives an example:

"We have very valid researchers in Italy; we have great Italian scientists operating in our laboratories and others worldwide." (M, fragment n. 7).

This affirmation is an exaltation of the value of Italian researchers. However, the president probably also intends to mention their difficulties finding a job in their country, often leading to the so-called "brain drain" issue.

Mattarella often refers to science in an idealized way. For example, it results when he talks about the need to make good use of discoveries. He frequently uses a metaphor of covid as a "referendum" on science. The Italian president affirms that vaccinations were a kind of referendum against science. The words "vaccine" and "referendum" point to this theme in the network about science (Fig 2). According to Mattarella, the referendum saw a result of "9 to 1 support of the benefits of science" (M, fragments n. 5 and n. 6). The referendum metaphor relates to the vaccination debate. In Italy, the anti-vaccine and no-mask minority mobilized on social media–[68] highlighted a sharp change in the emotions expressed about COVID-19 vaccines in trending Italian tweets, from joy and support to sadness and concern, after the (temporary) suspension of the AstraZeneca vaccine–and in the street during the pandemic.

These protests also echoed in the parliament. Mattarella has repeatedly expressed and supported his position favourable to vaccinations, indicating them as the instrument to overcome the crisis and relaunch the economy.

The next theme is science and civil liability. The Italian president often repeats how discoveries can also involve risks. To avoid such risks, he invokes a critical spirit, attentive to human lives, as in this fragment:

"Science is to help us with its enormous constant progress, but at the same time, the techniques operating to the borders of human life require a critical spirit in designing the future." (M, fragment n. 23).

Hence, the fear is that, alongside the great opportunities offered by the progress of science and technology, "new risks of homologation, exclusion, loss, distrust also emerge" (M, fragment n. 22). Based on this perspective, science needs to keep a high "sense of responsibility" (M, fragment n. 39). A continuous call to conscience (expressed by the word "conscience" in the network) and civic sense emerges. For example, in the case of the vaccine:

"It resulted from courageous choices, the progress of science, conscientious behaviours, diffused civic sense, and a convergence between institutions and citizens." (M, fragment n. 24).

Science also results as a powerful weapon. Unlike Johnson, however, it is not only an instrument to strengthen the country’s image. Instead, Mattarella points out that it will make the whole world better, even from a moral point of view, since "science offers us the strongest weapon, prevailing on ignorance and prejudice." (M, fragment n. 33). Speaking of the pandemic, which has hardly affected Italy, he finally states that vaccines should be "available for everyone worldwide" (M, fragment n. 35). This view, as opposed to Johnson’s, also connects to the universalistic role of science as a force that creates bridges and helps to overcome the barriers that separate peoples, as in this example:

"Research and science have conquered extraordinary results in such a short time, thanks to an international collaboration that has exceeded limits and barriers." (M, fragment n. 25).

### von der Leyen: The representation of science as a source of integration

The president of the European Commission frequently mentioned science in her speeches. However, she did less than Johnson and Mattarella. We found, in fact, 102 fragments out of 115 discourses with a fragments/speeches ratio of 0.89 (Table 1).

**Fig 3:**
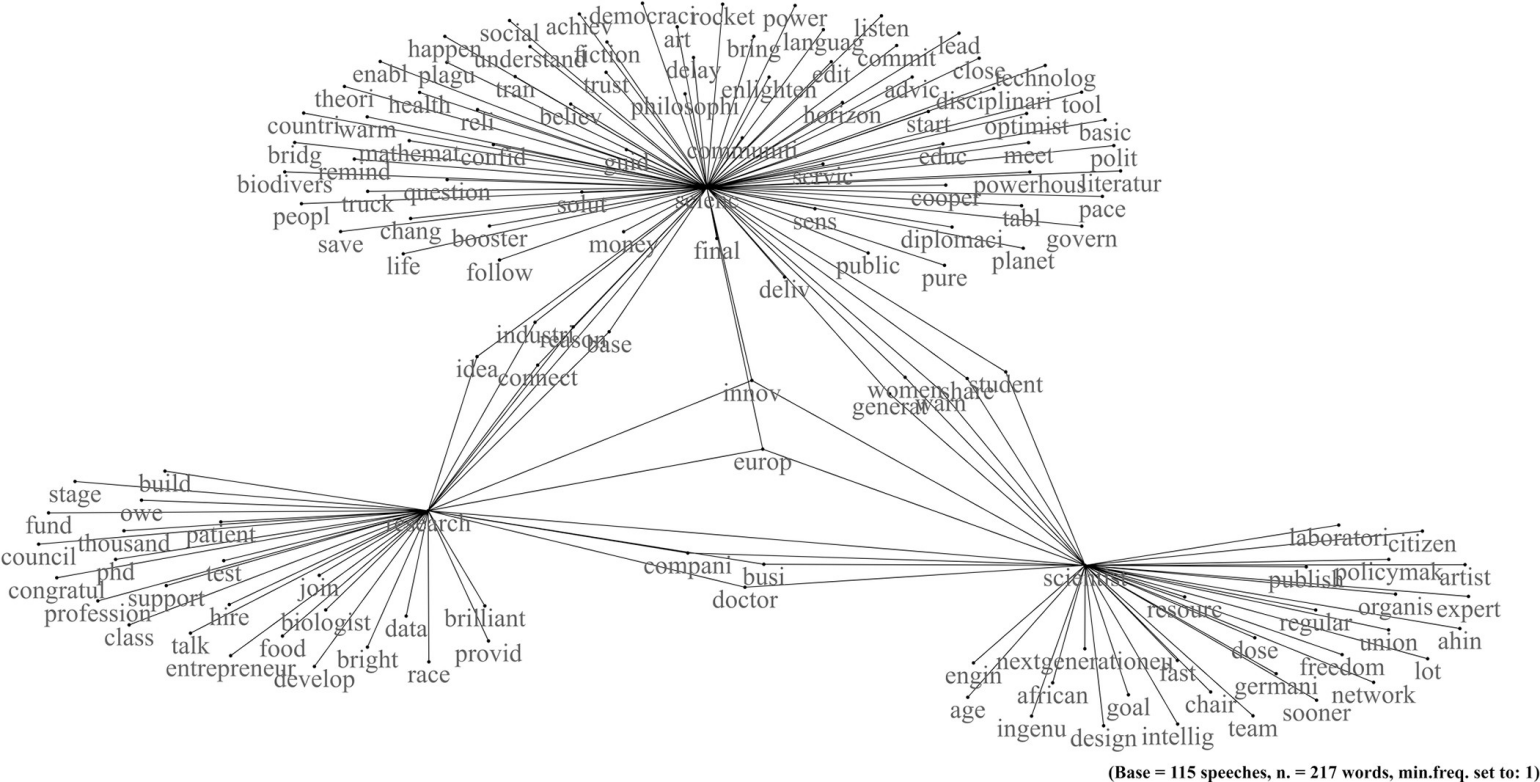
von der Leyen. Network of word co-occurrences about science.

The graphical representation of the co-occurrences shows three sub-networks centred around the words science, research and scientists (Fig 3). Two terms act as a bridge between the three networks. The first term is "Europe", which refers to the institutional level occupied by von der Leyen. The second is "innovation", which emerges, for example, from the following fragment where she affirms that the EU possesses this potential, in addition to the scientific ones: "we have the innovation and the scientific capacity—here […] and in the European Union "(vdL, fragment n. 13).

In her speeches, she touches on three major themes: the relationship of science with Europe, industry, and women’s rights.

Regarding the first theme, von der Leyen defines the European Union as the "powerhouse of science" (vdL, fragment n. 42) and Europe" links together the three networks (Fig 3). For this reason, she cites the tools that made it possible to achieve this position, such as the Horizon funding programme for scientific research (vdL, fragment n. 43). However, the concept is different from Johnson’s superpower, as it is broader and includes all EU countries. Here also comes into play the idea that science must lead to concrete results under Europe’s umbrella. An example is the following fragment where she affirms that innovations (the second word that unites the three networks) are the result of the encounter between science, ideas, and appropriate funding:

"[…] this is what Europe can achieve when finance meets science when ideas turn into innovation and products on the market". (vdL, fragment n. 45).

The second theme is the relationship with the industry. For von der Leyen, industry and science must work together to achieve success. Science should drive innovation—and, therefore, success—in the EU industry. The following excerpts are examples of this perspective: "Industry needs to keep up with science" (vdL, fragment n. 36) and "industry has to match the ground-breaking pace of science" (vdL, fragment n. 37). They differ significantly from Johnson’s idea, where the industry assigned the objectives to science. Here, the synergy also includes the relationship with institutions and cooperation between the public and the private sectors (vdL, fragment n. 40). Observing the network of word co-occurrences, for example, the fact that "industry" and "connect" unite the science and research networks confirms the link with the industries.

Finally, the third theme concerns women’s rights and gender equality and their role in science. The President of the European Commission enters the debate both as a woman—as she was the first woman to be elected president of the European Commission—and by her institutional role. Not surprisingly, "women" is one of the words that connect the sub-network of science with that of scientists (Fig 3). In her speeches, she firmly states that "Of course, women are made for science." (vdL, fragment n. 29) and "Some of Europe’s best scientists and engineers are women." (vdL, fragment n. 20). When she adheres more to her institutional role, the focus is instead on the fact that Europe seeks to promote the active participation of women in science since "It will invest in quality education for girls and women, including scientific education." (vdL, fragment n. 6).

Von der Leyen thus shows a multi-faceted vision of science linked to her institutional role. The analysis reveals a project where science, industry, and European institutions should work closely together to drive the economic and social well-being of the EU. Therefore, we have defined this perspective as an "integration" since, compared to Johnson and Mattarella, it aims at progress involving a plurality of stakeholders, and it is less anchored to national pride and the exaltation of ethical implications of science.

## Discussion

Since the beginning and throughout the pandemic crisis, science has been a key theme in speeches by political leaders. These speeches offer significant insights into the images and visions of science and its social and political role.

Three main narratives dominate the speeches analyzed in this study.

1. A *national pride* narrative that describes science as a key to strengthening the country’s position and influence in the international landscape. This narrative acquires, in some cases, a marked nationalistic tone, emphasizing national scientific achievements and presenting such achievements as a relevant indicator of the nation’s high international standing.
2. An *ethical* narrative that describes science as a carrier of positive values, e.g. passion, dedication to research work, social responsibility, and fights against ignorance and prejudice. These values and benefits of science are not just beneficial to a single country but to the international community.
3. An *integration* narrative that sees science as a driving force of economic, social development, and political identity. A strong relationship with the industry is a critical element of this narrative. However, science is also seen as a political resource to pursue aims like European integration, social inclusion, and gender equality. Science is also used in this narrative to define identity, particularly European identity, which has often been a critical point of European integration. In this light, she represents Europe as "the Powerhouse of Science".

Although different elements and themes are present in the individual speeches, each narrative is prevalent in the speeches of each of the three leaders. The *national pride* narrative is often recurrent in Johnson’s speeches; the *ethical* one is more evident in those by Mattarella; the *integration* narrative emerges more clearly in von der Leyen’s speeches.

This characterization can be easily associated with the different institutional and political contexts. In the case of Johnson, both the tradition of British Science and its relationship with governmental institutions and the more recent post-Brexit context play a role. Strong science is seen as an element of national pride and reaffirmation of national strength amid strong criticism and isolation concerns. The emphasis on national scientific experts and institutions can be thus seen as “a way of openly stating support and power at the same time” [28]. This is in line with the analysis conducted by [69] on national leaders’ political rhetoric during the COVID-19 pandemic, showing that Johnson and relied on upon—and fueled—“national fervor”, emphasizing national pride and reinforcing in-country identitiy and solidarity. As President of the Italian Republic, Mattarella has a representative and institutional role without specific governmental responsibility. Also, during the pandemic and in connection with changes in the government, he sought to provide institutional stability and continuity, emphasizing shared values and trust in science as a source of collective benefits. Specifically, this is in line with Mattarella’s recalls of European values and solidarity [49, 70]. Finally, the role of science and its achievements in fighting the pandemic crisis was seen by European institutions as an opportunity to strengthen and improve the perception of Europe and as a source of legitimation of relevant EU investments in research. In terms of broader responses to the COVID-19 pandemic, the mobilization of an integration narrative of science–through a clear and concise communication style [71]–highlights and supports the need for international collaboration to build a European Health Union to respond to cross-border health crises [72].

Political leaders can use their influence to address emergencies such as the COVID-19 pandemic [73], and their communication can influence people’s opinions and behaviours [74]. As the study has shown, an awareness of these narratives of science is essential both because a) they connect with key political narratives; b) they have influenced (and will probably continue to influence) policy decisions, not just about science and its funding but, more in general, about the role of science and scientific expertise in policy making. Compared with political leaders’ communication about and during the Covid-19 pandemic on social media platforms like Twitter (primarily focused on short-term, informative and moral boosting aims), these speeches offer a more articulated insight into the political narratives and visions of science’s role in society and politics [75].

We are, of course, aware of the limitations of our study as already mentioned above, and indeed we have no ambitions to consider them generalizable both in time and space, notably since we clearly stated our aim to study the presentation of science "in times of crisis". Further studies would be needed to analyze, for example, the relevance of these narratives outside Europe, how these narratives will change or adapt to future political contexts and new leaders and, more broadly, how science is represented in speeches by political leaders “at times of peace”, i.e. outside of crises and emergency contexts.

## Supporting information

S1 Appendix (DOCX)
