# Supplementary material for: The role of science in a crisis: Talks by political leaders during the COVID-19 pandemic
Source: PLoS One. 2023 Mar 24;18(3):e0282529. doi: 10.1371/journal.pone.0282529 (PMC10038249; doi:10.1371/journal.pone.0282529)
Supplement: S1 Appendix — (DOCX) [file pone.0282529.s001.docx]

**Appendix**

Table A1. Top keywords related to science and covid.

| Johnson | word | n. |
| --- | --- | --- |
|  | vaccin* | 300 |
|  | covid* | 178 |
|  | virus* | 156 |
|  | pandem* | 95 |
|  | variant* | 83 |
|  | scientist* | 45 |
|  | scienc* | 44 |
|  | scientif* | 39 |
|  | coronavirus* | 38 |
|  | innov* | 20 |
|  | nurs* | 13 |
|  | doctor* | 10 |
|  | research* | 1 |
| von der Layen |  |  |
|  | pandem* | 253 |
|  | innov* | 175 |
|  | covid* | 83 |
|  | scienc* | 66 |
|  | virus* | 62 |
|  | variant* | 29 |
|  | doctor* | 26 |
|  | scientist* | 26 |
|  | coronavirus* | 21 |
|  | research* | 21 |
|  | scientif* | 14 |
|  | nurs* | 8 |
| Mattarella |  |  |
|  | pandem* | 200 |
|  | virus* | 83 |
|  | innov* | 62 |
|  | scienc* | 62 |
|  | scientif* | 45 |
|  | covid* | 37 |
|  | doctor* | 29 |
|  | research* | 16 |
|  | coronavirus* | 12 |
|  | nurs* | 12 |
|  | scientist* | 12 |
|  | variant* | 3 |

le. A2. Networks of word co-occurrences: network statistics for scientific and covid related terms.

| Johnson | Degree | Betweenness centrality | Closeness centrality |
| --- | --- | --- | --- |
| coronavirus * | 95 | 80.0 | 0.0012009787 |
| covid* | 498 | 27853.0 | 0.0016252940 |
| doctor* | 36 | 809.6 | 0.0014111245 |
| innov* | 46 | 3.6 | 0.0011563511 |
| pandem* | 391 | 2960.0 | 0.0013328060 |
| scienc* | 210 | 901.0 | 0.0012797166 |
| scientif* | 108 | 123.0 | 0.0009004791 |
| scientist* | 55 | 0.0 | 0.0012781464 |
| vaccin* | 423 | 53508.0 | 0.0017695147 |
| variant* | 177 | 194.0 | 0.0013310866 |
| virus* | 353 | 3490.0 | 0.0013908170 |
| von der Layen |  |  |  |
| coronavirus* | 57 | 46.1 | 0.001823086 |
| covid* | 229 | 0.0 | 0.001177827 |
| doctor* | 15 | 0.0 | 0.001619572 |
| innov* | 273 | 626.0 | 0.001473104 |
| medic* | 60 | 392.0 | 0.001650012 |
| pandem* | 566 | 30742.0 | 0.001947799 |
| research* | 24 | 0.0 | 0.001585900 |
| scienc* | 82 | 85.0 | 0.001540649 |
| scientif* | 34 | 12.4 | 0.001534068 |
| scientist* | 70 | 75.0 | 0.001533377 |
| vaccin* | 510 | 12279.0 | 0.001784951 |
| variant* | 150 | 47.0 | 0.001428405 |
| virus* | 207 | 77.0 | 0.001223576 |
| Mattarella |  |  |  |
| covid* | 209 | 0.0 | 0.0009170904 |
| innov* | 93 | 383.0 | 0.0018205119 |
| pandem* | 407 | 9106.0 | 0.0018837685 |
| research* | 31 | 74.6 | 0.0017928478 |
| scienc* | 180 | 166.0 | 0.0015494221 |
| scientif* | 221 | 13.0 | 0.0013452382 |
| vaccin* | 295 | 415.0 | 0.0015139670 |
| virus* | 200 | 270.0 | 0.0015000982 |
